# Enhancing knowledge of vascular pythiosis: Impact of a self-paced online course among Thai learners

**DOI:** 10.1371/journal.pntd.0013003

**Published:** 2025-04-08

**Authors:** Pattama Torvorapanit, Surachai Leksuwankun, Rongpong Plongla, Navaporn Worasilchai, Nattapong Langsiri, Ariya Chindamporn, Achitpol Thongkam, Nuttapon Susaengrat, Pongsakorn Ouwongprayoon, Karan Srisurapanont, Kasama Manothummetha, Nipat Chuleerarux, Tanaporn Meejun, Jaedvara Thanakitcharu, Bhoowit Lerttiendamrong, Nitipong Permpalung, Saman Nematollahi

**Affiliations:** 1 Thai Red Cross Emerging Infectious Diseases Clinical Center, King Chulalongkorn Memorial Hospital, Bangkok, Thailand; 2 Department of Medicine, Division of Infectious Diseases, Faculty of Medicine, Chulalongkorn University, and King Chulalongkorn Memorial Hospital, Thai Red Cross Society, Bangkok, Thailand; 3 Department of Medicine, Faculty of Medicine, Chulalongkorn University, and King Chulalongkorn Memorial Hospital, Thai Red Cross Society, Bangkok, Thailand; 4 Center of Excellence in Antimicrobial Resistance and Stewardship, Chulalongkorn University, Bangkok, Thailand; 5 Department of Transfusion Medicine and Clinical Microbiology, Faculty of Allied Health Sciences, Chulalongkorn University, Bangkok, Thailand; 6 Research Unit of Medical Mycology Diagnosis, Chulalongkorn University, Bangkok, Thailand; 7 Department of Microbiology, Faculty of Medicine, Chulalongkorn University, Bangkok, Thailand; 8 Department of Surgery, Khonkaen Hospital, Khonkaen, Thailand; 9 Department of Radiology, Division of Diagnostic Radiology, Faculty of Medicine, Chulalongkorn University and King Chulalongkorn Memorial Hospital, Bangkok, Thailand; 10 Department of Medicine, Faculty of Medicine, Chiang Mai University, Chiang Mai, Thailand; 11 Department of Medicine, Jackson Memorial Hospital/University of Miami, Miami, Florida, United States of America; 12 Department of Pediatrics, Panyananthaphikkhu Chonprathan Medical Center, Srinakharinwirot University, Nonthaburi, Thailand; 13 Faculty of Medicine, Siriraj Medical Simulation for Education and Training, Siriraj Hospital, Mahidol University, Bangkok, Thailand; 14 Department of Medicine, Johns Hopkins University School of Medicine, Baltimore, Maryland, United States of America; 15 Department of Medicine, University of Arizona College of Medicine, Tucson, Arizona, United States of America; Albert Einstein College of Medicine, UNITED STATES OF AMERICA

## Abstract

**Background:**

Pythiosis caused by *Pythium insidiosum*, is a rare but deadly infectious disease that is often underrecognized. The disease has high morbidity and mortality rates, particularly in vascular forms where surgical resection is necessary. A previous study demonstrated low awareness and knowledge of vascular pythiosis among Thai medical personnel. There is an urgent need to improve disease recognition given that vascular pythiosis is very prevalent in Thailand.

**Methods:**

This study aimed to enhance knowledge and disease recognition about vascular pythiosis among Thai medical personnel and the public through a self-paced, asynchronous, open-access online course. The course included seven video lessons and was available from February to July 2023. Participants’ knowledge was assessed using pretest and posttest analysis.

**Results:**

A total of 428 participants completed the course. Participants showed significant knowledge improvement, with mean posttest scores substantially higher than pretest scores, 6.77 vs. 3.46 (p-value < 0.01). Higher educational level had a positive impact with higher pretest and posttest scores, but the delta scores between the posttest and pretest of all groups were comparable. Moreover, 80% of participants demonstrated knowledge gain. However, participants of all groups scored the lowest on the posttest in diagnostic investigation field.

**Conclusions:**

Very low pretest scores underline the neglected problem of vascular pythiosis. This asynchronous online course successfully enhanced participants’ knowledge about vascular pythiosis. Future efforts should focus on collaborative initiatives at the government and university levels to emphasize disease recognition.

## Author summary

Human pythiosis is an infectious disease caused by a fungal-like organism, *Pythium insidiosum*. The vascular form is the deadliest manifestation compared to cutaneous and ocular pythiosis. Thailand has reported the highest number of vascular pythiosis cases globally. However, vascular pythiosis remains primarily recognized by medical personnel in specialized fields. Achieving a definitive diagnosis remains challenging, and rapid diagnostic tests are currently under the research. This reflects a bidirectional dilemma—diagnostic challenges contribute to high case fatality rates, while under-recognition further delays diagnosis. This study aimed to enhance disease recognition and knowledge of vascular pythiosis among both medical professionals and the general public in Thailand through a self-access online course, conducted by experts with clinical and research experiences in the disease. The online course serves as a foundation step in addressing the knowledge gap and establishing an educational resource on vascular pythiosis in Thailand. Knowledge gain was assessed through a pretest-posttest comparison. The results highlighted the issue of neglect and provided valuable data to support the development of future educational initiatives, with contents adaptable to different target audiences.

## Introduction

Despite their rarity, fatal infectious diseases must be recognized by both healthcare professionals and the public. Pythiosis, caused by *Pythium* spp*.*, a fungal-like pathogen, has been infrequently reported worldwide, with only 771 documented cases from 1980 to 2021 [1]. The primary species responsible for human infection is *Pythium insidiosum*, which presents in four main clinical forms: ocular, cutaneous, vascular, and disseminated infections. Treatment protocols are not yet standardized, often necessitating extensive surgical intervention and prolonged antimicrobial therapy, leading to significant morbidity and mortality. For vascular pythiosis, studies indicate a nearly 100% mortality rate in cases where complete surgical resection is not achieved [2–4]. Recently, our study team conducted the first clinical trial on vascular pythiosis, demonstrating improved survival rates with a combination of surgery with azithromycin, doxycycline and itraconazole for at least 6 months, continuing until serum 1,3 beta-d-glucan became negative for those with residual disease [5].

However, in Thailand, which accounts for the highest number of vascular pythiosis cases globally, awareness among general physicians is concerningly low, with only 30% familiar with the disease and just 15% knowledgeable about the necessary diagnostic investigations [6]. This limited awareness underscores an urgent need to enhance understanding of vascular pythiosis, particularly in Thailand, to improve disease recognition and early intervention.

An open-access, self-paced online course offers an ideal format for disseminating this knowledge, catering to individuals with varying levels of familiarity with the topic and enabling flexible access to critical information. In this study, we aim to improve awareness and understanding of vascular pythiosis among healthcare professionals and the public through a structured, open-access online educational course.

## Methods

### Ethics statement

Ethical approval for the study was granted in accordance with the Declaration of Helsinki by the Institutional Review Board of the Faculty of Medicine, Chulalongkorn University (COE 033/2022).

### Research aims and design

Our goal was twofold: to equip medical providers with the ability to recognize vascular pythiosis and initiate prompt investigations and management, and to educate the public on its signs and symptoms so they can seek medical attention promptly. The primary objective of this study was to improve awareness and knowledge regarding the disease manifestations among healthcare professionals and the public in Thailand. Secondary objectives included improving understanding of diagnostic investigations, proper handling of clinical specimens, and the timing of surgical and medical management. While these secondary objectives were open to all interested participants, they were primarily designed for healthcare providers. To achieve this, we implemented an asynchronous, open-access online course, accessible to participants from February to July 2023. This study employed a cross-sectional one-group pretest-posttest design to evaluate baseline knowledge and measure knowledge improvement across participants with varying educational backgrounds following course completion.

### Operational terms

“Quiz” refers to the assessment after each lesson evaluating comprehension and retention of the material. “Course Completion” is defined as the completion of over 80% of course content, including both the pretest and the posttest. The term “< BC” (below bachelor degree of education) indicates individuals currently pursuing undergraduate studies, including medical students in Thailand. “BC” (bachelor degree of education) refers to individuals who have completed undergraduate studies and those currently engaged in postgraduate studies, including medical residency trainees in Thailand, and “> BC” (above bachelor degree of education) represents those who have completed postgraduate studies.

### Setting and participants

The online course, titled “Vascular Pythiosis, Deadly Tropical Disease: Diagnosis and Current Treatment,” was hosted on the “MyCourseVille (MCV)” learning platform, operated by the “CHULA MOOC” project of Learning Innovation Center, Chulalongkorn University, Bangkok, Thailand. Course promotion occurred one month prior to its launch, targeting both the general public and medical professionals through the MCV website (https://www.mycourseville.com/?q=onlinecourse), and Facebook page of CHULA MOOC (https://www.facebook.com/CHULAMOOC/). MCV is offered to anyone who is interested in learning with an online platform and not restricted to students or staff of Chulalongkorn University. Moreover, the course was also promoted by the Facebook page of Mycology, Epidemiology, and Medical Education Research Group (MERG) (https://www.facebook.com/MERGpythiosis), which is operated by our study team. CHULA MOOC and MERG Facebook pages have 234,000 and 255 followers, respectively. Participants registered for the course free of charge. Each registrant was assigned a unique participant ID used for course access and tracking progress throughout the study.

### Educational intervention

The course consisted of asynchronous lectures based on a prior needs assessment survey that identified knowledge gaps in vascular pythiosis [6]. Seven video lectures were developed by experts with extensive clinical and research experience in vascular pythiosis in Thailand. The lectures presented information in a narrative review and case-based format on topics including epidemiology, clinical presentation, microbiology, specimen collection, imaging studies and current treatment options. Each lecture was delivered in Thai, with Thai subtitles, and had a duration of 10–20 minutes per lesson. The total course duration is roughly 120 minutes. Participants were able to engage with the course content at their own pace over a 6-month course period, with no restrictions on the order or timing of lessons. No financial incentives were provided to participants. Certificates of completion were issued automatically by MCV upon achieving passing scores without monetary benefit or education credits. The course syllabus which includes objectives, target learning outcomes and exam blueprint, and exam questions are available in S1 File.

### Evaluation

Participants were required to complete a pretest prior to accessing the course content. Following each lesson, a quiz assessed knowledge retention. Participants could attempt each quiz multiple times, with only the final attempt recorded; however, correct answers were not displayed. Course progression was monitored, and participants were required to complete over 80% of the course content to access the posttest and successfully complete the course. Knowledge gain was assessed by comparing pretest and posttest scores. Each participant had only one attempt for both the pretest and posttest. A final course score was computed by combining 50% from the cumulative quiz scores and 50% from the posttest score, with a passing threshold of 60% for certificate eligibility.

The quiz, pretest, and posttest questions were developed by a team of four experts: two specialists in ‘Infectious Diseases’ and two in ‘Medical Education’. This collaborative effort ensured comprehensive assessment and alignment with the course’s learning objectives. Each quiz contained three questions selected from a pool of five, while the pretest and posttest comprised twelve identical questions drawn from all lessons. The order of questions and answer choices were randomized to ensure variability across assessments.

### Data analysis

All registered participants who completed the course were included in the analysis. Individual consent was not obtained because of anonymity. No sample size calculation was performed. Statistical analysis was performed using R program (version 4.4.1; R foundation for Statistical Computing, Vienna, Austria). Data were retrieved from the Chulalongkorn Learning Innovation Center system using unique participant ID numbers, ensuring that identities remained confidential to researchers. For descriptive statistics, categorical data were presented as frequencies (n) and percentages (%), while continuous data were presented as medians (interquartile range; IQR). For the proportion as percentages, its central tendency was presented by means ± standard deviation; SD. A paired Wilcoxon Rank Sum test was used to analyze the scores between the pretest and posttest. The Kruskal-Wallis test was performed to examine differences in knowledge gain across education levels (< BC, BC and > BC), with significant differences indicated by a p-value < 0.05.

## Results

### Participant enrollment

**Fig 1:**
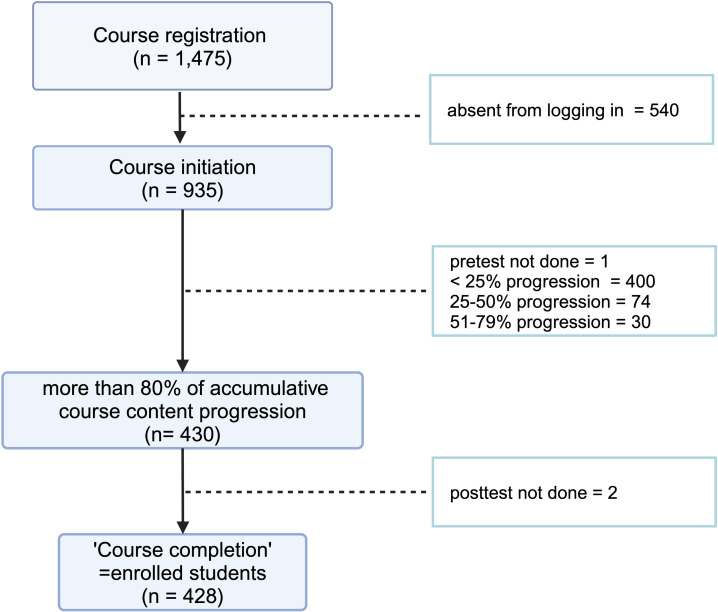
Scheme of enrolled participants. Fig 1 was created in BioRender. Torvorapanit, P. (2025) https://BioRender.com/g00h497.

Throughout the course period, a total of 1,475 individuals registered, of whom 935 logged in and initiated the course. A total of 430 (45.99%) participants were eligible to take the posttest by completing more than 80% of the course content. Of these, 428 (99.5%) participants completed the posttest and were subsequently included in the study analysis (Fig 1).

### Demographic and learning characteristics of the enrolled participants

The 428 participants included in the analysis had a median age of 19 years (interquartile range (IQR)18–31). Most participants were females (60.75%) and currently enrolled in educational programs such as high school, college, residency or fellowship program (80.37%). Additionally, 173 (40.42%) of participants resided in Bangkok, Thailand. Educational levels were categorized as followed: 332 (77.57%) participants were pursuing education below a bachelor’s degree (< BC), 71 (16.59%) participants had attained a bachelor’s degree (BC), and 25 (5.84%) participants held qualifications above a bachelor’s degree (> BC). On average, participants completed 98.41% (mean ± SD 3.15) of the course. Three-hundred and seventy-seven (88.08%) participants completed the course and received a certificate of accomplishment (Table 1).

**Table 1:** Characteristics of enrolled participants by group of educational level.

Characteristics	Total (n = 428)	< BC (n = 332)	BC (n = 71)	> BC (n = 25)
Age (median (IQR); years)	19 (18–31)	19 (18–19)	31 (26–34)	38 (33–45)
Gender (n (%))				
• Male	109 (25.47%)	70 (21.08%)	30 (42.25%)	9 (36.00%)
• Female	260 (60.75%)	208 (62.65%)	37 (52.10%)	15 (60.00%)
• Not specified	59 (13.79%)	54 (16.27%)	4 (5.63%)	1 (4.00%)
Occupation (n (%))				
• Student	344 (80.37%)	315 (94.88%)	22 (30.99%)	7 (28.00%)
• Business owner	12 (2.80%)	5 (1.50%)	7 (9.86%)	0 (0.00%)
• Government employee	32 (7.48%)	3 (0.90%)	18 (25.35%)	11 (44.00%)
• Private sector employee	12 (2.80%)	3 (0.90%)	9 (12.68%)	0 (0.00%)
• Unemployed	8 (1.87%)	1 (0.30%)	5 (7.04%)	2 (8.00%)
• Not specified	20 (4.67)	5 (1.50%)	10 (14.08%)	5 (20.00%)
Address area (n (%))				
• Bangkok	173 (40.42%)	122 (36.75%)	41 (57.75%)	10 (40.00%)
• Central	46 (10.75%)	38 (11.45%)	5 (7.04%)	3 (12.00%)
• Northern	32 (7.48%)	28 (8.43%)	2 (2.82%)	0 (0.00%)
• Northeast	78 (18.22%)	58 (17.47%)	12 (16.90%)	10 (40.00%)
• Southern	59 (13.79%)	55 (16.57%)	4 (5.63%)	0 (0.00%)
• Others in Thailand	37 (8.64%)	29 (8.73%)	6 (8.45%)	2 (8.00%)
• Out of Thailand	3 (0.70%)	2 (0.60%)	1 (1.41%)	0 (0.00%)
Course progression (% proportion) (mean ± SD)	98.41± 3.15	98.23 ± 3.29	98.94 ± 2.58	99.26 ± 2.37
Certificate completion (n (%))	377 (88.08%)	286 (86.14%)	68 (95.77%)	23 (92.00%)

Abbreviations; < BC: below bachelor degree, BC: bachelor degree and > BC: above bachelor degree

### Recorded scores and analysis

Across all educational groups, participants achieved high quiz scores, though the number of quiz attempts was not recorded. The median final summative quiz scores were 21 (IQR 20–21) out of a possible 21 points for all groups (Table A in S1 File). The median posttest score, out of a maximum of 12, was significantly higher than the pretest score (7 vs. 3, p < 0.001). A positive association was observed between educational level and median scores; with pretest scores of 3, 3, and 5, and posttest scores of 7, 8, and 10 for the < BC, BC, and > BC groups, respectively (Table 2).

**Table 2:** Pretest and posttest score analysis by group of educational level.

Participants	Pretest median score (IQR)	Posttest median score (IQR)	p-value*
< BC (n = 332)	3 (2–4)	7 (5–8)	< 0.001
BC (n = 71)	3 (2–5)	8 (6–9)	< 0.001
> BC (n = 25)	5 (3–9)	10 (7–11)	< 0.001
Total (n = 428)	3 (2–5)	7 (5–9)	< 0.001

Abbreviations; < BC: below bachelor degree, BC: bachelor degree and > BC: above bachelor degree

*Significant p-value for difference across group by Kruskal Wallis H test was < 0.05

Posttest scores were also analyzed across five domains of vascular pythiosis knowledge: epidemiology, microbiology and clinical syndrome, diagnostic investigations, sample collection and handling, and treatment. The mean posttest scores for these domains were 67.17%, 67.76%, 36.16%, 74.88%, and 56.43%, respectively (Table 3).

**Table 3:** Percentage of posttest scores stratified by knowledge domain about vascular pythiosis among three groups.

Posttest score (%; mean ± SD)	Total (n = 428)	< BC (n = 332)	BC (n = 71)	> BC (n = 25)
Epidemiology	67.17 ± 33.75	65.66 ± 34.70	69.72 ± 31.03	80.00 ± 25.00
Microbiology and clinical syndrome	67.76 ± 37.80	64.46 ± 39.28	76.76 ± 31.43	86.00 ± 22.91
Diagnostic investigations	36.16 ± 26.55	33.81 ± 24.73	39.79 ± 29.15	57.00 ± 32.69
Sample collection and handling	74.88 ±36.62	72.44 ± 37.70	82.40 ± 31.73	86.00 ± 30.69
Management	56.43 ±39.16	50.90 ± 38.07	71.13 ± 38.42	88.00 ± 29.86

Abbreviations; < BC: below bachelor degree, BC: bachelor degree and > BC: above bachelor degree

**Fig 2:**
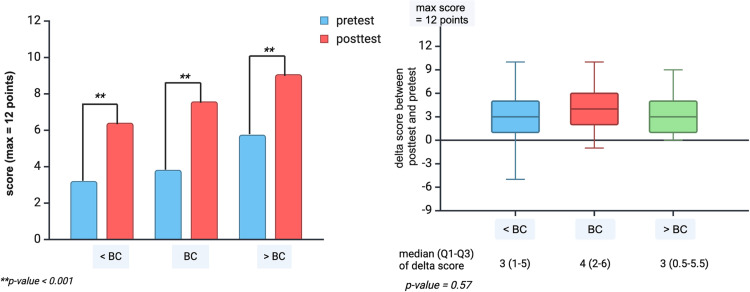
A (left) pretest and posttest scores and B (right) the delta scores between posttest and pretest among three groups. Both graphs were created in https://BioRender.com, which allows usage with acknowledgment. Abbreviations; < BC, below bachelor degree of education; BC, bachelor degree of education; and > BC, above bachelor degree of education.

A statistically significant increase in posttest scores compared to pretest scores was observed across all three educational groups (Fig 2A). However, the median (IQR) of the delta score between the posttest and pretest was 3 (1–5) for the < BC group, 4 (2–6) for the BC group, and 3 (0.5-5.5) for the > BC group, which did not demonstrate a significant difference among all groups (p-value = 0.57) (Fig 2B).

Knowledge improvement, represented by a positive delta score, was observed in 267 (80.42%) participants in the < BC group, 60 (84.51%) participants in the BC group, and 19 (76%) participants in the > BC group. The remaining participants either scored lower or showed no improvement on the posttest compared to the pretest.

## Discussion

This study aimed to enhance disease recognition and improve knowledge of diagnosis and treatment of vascular pythiosis among the Thai population. To our knowledge, this is the first widely accessible and comprehensive learning resource on vascular pythiosis available to the public. The participants were registered from all regions in Thailand, with three individuals accessing the course from outside of Thailand. Of those who completed the course, approximately 80% demonstrated measurable knowledge gains based on posttest scores.

The incidence of human pythiosis is likely underestimated due to limited access to diagnostic tests and a lack of familiarity with the disease among healthcare providers. Fewer than 40 cases are reported globally each year, with most cases documented only since 2008 [1]. For vascular pythiosis, nearly all cases worldwide are reported from Thailand, where a previous retrospective cohort estimated an average national incidence of three cases per year [7]. However, our recent trial, registered with the Thai Clinical Trials Registry (TCTR) on 17^th^ December 2019 (Clinical Trial Number TCTR20191217006), enrolled 10–20 new cases of vascular pythiosis annually, highlighting the urgent need for increased disease awareness and enhanced diagnostic protocols [5]. Limited clinical exposure to vascular pythiosis challenges primary care physicians, who may hesitate to diagnose or request relevant diagnostic investigations. Unpublished 2023 survey data from our team found that 79.3% (65/82) of non-medical personnel were unfamiliar with pythiosis. This knowledge gap might contribute to a cycle of under-recognition and delayed diagnosis.

There is ongoing debate about the effectiveness of teaching complex topics through online platforms in terms of enhancing knowledge. Using MOOCs (Massive Open Online Courses) is suitable for broadening general knowledge but not for complicated skill training [8,9]. However, a significant benefit of MOOCs is that they allow learners to study at their convenience, free from time constraints, making it ideal for widespread education. For infectious diseases content, prior studies also resulted in controversial impact. Wang et al. demonstrated that asynchronous e-learning had a positive impact on TB knowledge improvement among primary care workers in China [10]. Another study in India demonstrated that video-assisted teaching significantly increased posttest scores about COVID-19 disinfection, but not for personal protective equipment practice [11]. Since our study primarily aimed to increase awareness and understanding of a disease, a self-paced, open-access online course was an appropriate and beneficial option.

Our course promotion conducted through the website attracted over 1,000 participants, possibly due to increased interest in infectious diseases in the post-COVID-19 era. Additionally, the course being free of charge contributed to its initial appeal. However, this broad course promotion attracts a diverse target audience. A significant number (36.6%) of participants did not start the course. Among those who did start, only about half completed it. This low engagement may be attributed to a lack of true interest in the topic from the outset, the content or teaching style not meeting their needs, lack of understanding of the material, having other competing engagements (e.g., schoolwork, job, etc.), or having no financial incentive. The low engagement has been noted to be a common problem for MOOC delivery [12].

The knowledge outcomes were analyzed based on different educational levels. For those who completed the course, it was observed that educational level positively affected both pretest and posttest scores. Interestingly, the delta scores were similar among all groups. This might imply that our online course has a consistent impact of knowledge gain for all participants, but the overall knowledge acquisition depends on the participants’ background educational level. The > BC group may have had a higher proportion of healthcare providers, which could have led to higher scores in that group. However, in the groups < BC and BC, some participants’ scores decreased. This could be explained by some individuals attending the course merely to pass time or obtain a certificate with non-specific purpose but no active engagement.

For knowledge of vascular pythiosis categorized by learning objectives, we allowed multiple quiz attempts after each lesson to ensure participants can select the correct answer. There were high quiz scores for all participants among all lessons. The final assessment of knowledge gain was demonstrated by the posttest. We found that participants had the highest percentage of posttest scores in sample collection and handling, with 74.88% ± SD 36.62. The lowest percentage of posttest scores was in diagnostic investigations, with 36.16% ± SD 26.55. Educational level also impacted the posttest scores for all knowledge domains. However, the > BC group scored only 57% ± SD 32.69 for diagnostic investigations, compared to over 80% for other domains. This might imply that competency in choosing investigations and interpreting results requires more clinical experience beyond lectures.

Our study had some limitations. First, we lacked definitive data to differentiate between general participants and healthcare professionals, which potentially limited our ability to assess the program’s effectiveness. Second, non-response bias was a concern, as a significant proportion of individuals did not complete the course. This may indicate that we selected participants with specific interests or learning purposes. Third, we did not monitor participants’ screen time during the learning process, which could have affected the quality of learning and knowledge retention.

This work will guide the development of an improved version of the course. Developing an English soundtrack or subtitles will help expand the course’s accessibility to non-Thai participants. Dividing the course into two sections—one for general participants and another for healthcare providers—will make it easier to tailor content for each group. Given the low posttest scores in diagnostic interventions (36.16%) and management (56.43%), future courses can focus on these two areas. Particularly for healthcare providers, we plan to create separate modules and workshops for specific groups, such as emergency medicine, vascular surgery, and microbiology. Moreover, the development of rapid test kits might be a promising option alongside the educational effort [13], along with collaboration at the government and university levels to further combat the neglect of vascular pythiosis.

## Conclusion

The very low pretest scores highlight the significant issue of disease under-recognition. Our online course demonstrated knowledge gain across the country for all participants. Our study marks a vital first step in creating a comprehensive learning resource on human vascular pythiosis in Thailand and might be the basis to support uncovering vascular pythiosis cases in the future.

## Supporting information

S1 File Supplemental materials. (DOCX)

S2 File Supporting data. (PDF)
