# Supplementary material for: Enhancing knowledge of vascular pythiosis: Impact of a self-paced online course among Thai learners
Source: PLoS Negl Trop Dis. 2025 Apr 8;19(4):e0013003. doi: 10.1371/journal.pntd.0013003 (PMC12005490; doi:10.1371/journal.pntd.0013003)
Supplement: S1 File — (DOCX) [file pntd.0013003.s001.docx]

**Supplemental materials**

**Course Syllabus (version 1.0, 7th February 2023)**

1. **Instructor Responsible for the Course:**
   Name: Dr. Pattama T. Worapanit
   Department/Division:

- Clinical Infectious Disease Center, King Chulalongkorn Memorial Hospital, Thai Red Cross Society
- Division of Infectious Diseases, Department of Medicine, Faculty of Medicine, Chulalongkorn University
  Phone: 089-239-3400
  Email: pattama.t@chula.ac.th

1. **Course Coordinator:**
   Name: Mr. Nattapong Lengsiri
   Department/Division:

- Department of Microbiology, Faculty of Medicine, Chulalongkorn University
  Phone: 093-428-7977
  Email: pythiosisonlinelearning@gmail.com

1. **Course Title:**
   (Thai) การวินิจฉัยและการรักษาโรคพิธิโอซิสในหลอดเลือดในปัจจุบัน
   (English) Vascular Pythiosis Diagnosis and Current Treatment
2. **Course Content:**

- Epidemiology and clinical presentation of pythiosis
- Characteristics and identification of *Pythium insidiosum*
- Microbiological testing procedures
- Radiologic testing and interpretation
- Evidence-based management of vascular pythiosis
- Case studies of vascular pythiosis
- Monitoring treatment response and drug side effects
- Role of surgery
- Consulting with experts

1. **Course Objectives:**

**5.1 Primary objectives**
5.1.1 Highlight the significance of vascular pythiosis in Thailand and describe key disease features to healthcare professionals and general public.

**5.2 Secondary objectives**
5.2.1 Develop clinical skills in diagnosing vascular pythiosis among medical students and general practitioners.
5.2.2 Provide up-to-date evidence-based patient management information.

1. **Learning Outcomes:**
   By the end of the course, learners will be able to:
   1. Describe the epidemiology of pythiosis

6.2 Identify key clinical symptoms

6.3 Explain microbiological characteristics of *Pythium insidiosum*

6.4 Describe microbiological testing processes

6.5 Interpret radiologic results

6.6 Explain treatment methods

6.7 Discuss disease progression and treatment follow-up

6.8 Identify expert consultation channels

1. **Course Topics and Instructors:**

- Introduction to the course (Various Instructors) (5 minutes)
- Epidemiology and clinical presentation (Dr. Pattama Torvorapanit) (20 minutes)
- Pythiosis in Microbiology (Assoc. Prof. Dr. Ariya Chindamporn) (15 minutes)
- Specimen Collection and Laboratory Findings (Dr. Nawaporn Worasilchai) (12 minutes)
- Radiologic Interpretation (Dr. Pongsakorn Uawongprayoon) (20 minutes)
- Evidence-based Management (Asst. Prof. Dr. Nitipong Permpalang) (13 minutes)
- Case-based Learning: Clinical Diagnosis (Dr. Rongpong Plongla) (20 minutes)
- Case-based Learning: Surgical Role (Dr. Nattapon Susangrat) (10 minutes)

1. **Target Learners:**
   8.1 Medical students and physicians
   8.2 Other healthcare professionals
   8.3 General public
2. **Total Learning Hours:**
   120 minutes
3. **Teaching Methods:**

- Asynchronous Online Learning: via MyCourseVille platform operated by CHULA MOOC program, learning innovation center, Chulalongkorn University, Bangkok, Thailand

1. **Evaluation Methods:**

- Summative assessments using single best answer format

1. **Course completion and Certification Criteria:**
   12.1 Learners complete the course when passing 80% of all course contents will be allowed to access the posttest evaluation.
   12.2 Learners receive certification from CHULA MOOC upon scoring at least 60% on the final evaluation, which is calculated from the summative quiz scores and the posttest, each contributing 50% to the final score.
2. **Instructional Media:**

- Recorded lectures
- Animations, motion graphics, and infographics

1. **Exam Blueprint:**

| **Test/Quiz** | **Learning outcomes** | **Number of questions** |
| --- | --- | --- |
| Pretest and posttest | 6.1-6.8 | 12 |
| Quiz 1 | 6.1, 6.2 | 3 |
| Quiz 2 | 6.3 | 3 |
| Quiz 3 | 6.3, 6.4 | 3 |
| Quiz 4 | 6.5 | 3 |
| Quiz 5 | 6.6, 6.8 | 3 |
| Quiz 6 | 6.6, 6.7 | 3 |
| Quiz 7 | 6.6, 6.7 | 3 |

**The examination**

**Pretest and posttest (n=12)**

Domain 1. Epidemiology

1.1 In cases of pythiosis infection, which organ is most commonly affected worldwide?

A. Eye
B. Skin
C. Blood vessels
D. Lungs
E. Multiple organs

1.2 In cases of pythiosis infection reported in Thailand, which organ is most commonly affected?

A. Eye

B. Skin

C. Blood vessels

D. Lungs

E. Multiple organs

Domain 2. Microbiology and clinical syndrome

2.1 What is the infectious stage of *Pythium insidiosum* that adheres to the patient's wound?

A. Hyphae

B. Encysted zoospores

C. Zoospores with flagella

D. Secreted products

E. Flagella part

2.2 Which of the following is correct regarding vascular pythiosis?

A. Surgery does not require clear margins

B. *Pythium insidiosum* is a type of fungus

C. Vascular pythiosis in the leg can be treated with medication alone once the wound starts healing

D. Iliac artery pythiosis cannot be surgically treated

E. Vascular pythiosis can present with both acute and chronic arterial occlusion

Domain 3. Diagnostic investigations

3.1 Which radiologic tool is most suitable for assessing the severity and extent of vascular pythiosis?

A. Plain radiograph

B. Ultrasound

C. Plain MDCT scan

D. Contrast-enhanced MDCT scan

E. PET/CT scan

3.2 In vascular pythiosis patients, which vascular abnormality is least commonly found?

A. Arterial occlusion

B. Aneurysm

C. Arteritis or phlebitis

D. Atherosclerosis

E. Ruptured pseudoaneurysm

3.3 A 40-year-old male diagnosed with pythiosis of the right femoral artery 6 months ago, treated with surgery and antifungal medication, presents with severe pain at the surgical site and low-grade fever. Pathology showed no residual infection. What should be the first step?

A. Blood culture

B. Galactomannan test

C. X-ray of the surgical site

D. CT angiogram

E. Antifungal drug level testing

3.4 A 55-year-old male farmer has chronic left leg pain for 3 months, worsening with walking. Examination shows left leg atrophy, hair loss, shiny skin with old scars, and diminished pulses in the left popliteal, dorsalis pedis, and posterior tibial arteries. Which action is NOT appropriate?

A. Administer heparin

B. Test for *Pythium* antibodies

C. Perform doppler ultrasound

D. Conduct CT angiogram

E. Biopsy old scar tissue for culture

Domain 4. Sample collection and handling

4.1 In vascular pythiosis, which patient specimen is most likely to yield a positive culture result?

A. Arterial wall

B. Thrombus in the artery

C. Blood sample

D. Tissue sample from lesion site

E. Skin from lesion site

4.2 Which specimen type and transport method are most appropriate if transportation takes more than 4 hours to diagnose vascular pythiosis?

A. Send arterial wall in a sterile container without freezing

B. Send thrombus in a sterile container with freezing

C. Send whole blood in a sterile blood collection tube with freezing

D. Send plasma/serum in a sterile blood collection tube with freezing

E. Send skin sample from the lesion site in a sterile container without freezing

Domain 5. Management

- 1. A 50-year-old female diagnosed with pythiosis in the right popliteal artery 4 months ago, treated with surgery and antifungal drugs, continues to have pain at the surgical site and elevated beta-D-glucan levels (>500 pg/ml). Imaging reveals new thrombus in the right femoral artery. What treatment adjustment will improve survival the most?

A. Add amphotericin
B. Add micafungin
C. Add vancomycin and meropenem
D. Administer *Pythium insidiosum* vaccine
E. Additional surgery to remove infected tissue

- 1. Which antibacterial drug class can be used to treat vascular pythiosis?

A. β-lactam
B. Aminoglycoside
C. Glycopeptide
D. Fluoroquinolone
E. Macrolide

**Quiz 1 (n=3)**

1. The first confirmed case of pythiosis in the world was reported from which country? A. India
   B. Australia
   C. Brazil
   D. Thailand
   E. United States
2. In cases of pythiosis infection reported in Thailand, which organ is most commonly affected?

A. Eye
B. Skin
C. Blood vessels
D. Lungs
E. Multiple organs

1. A Thai woman, 35 years old, working as a tour guide from Chiang Rai, a thalassemia carrier, returned from a pilgrimage in India. She swam in the Ganges River 2 weeks prior and developed worsening right eye redness and blurred vision. Which history detail most supports a differential diagnosis of ocular pythiosis?

A. Female gender
B. Tour guide occupation
C. Chiang Rai residency
D. Thalassemia carrier status
E. Swimming in the Ganges River

**Quiz 2 (n=3)**

1. What is the natural aquatic form of *Pythium insidiosum*?

A. Hyphae with regular septa
B. Hyphae with few septa
C. Dark-colored spore-producing hyphae
D. Hyphae and zoospores
E. Hyphae and chlamydospores

1. What is the infectious stage of *Pythium insidiosum* that adheres to patient wounds? A. Hyphae
   B. Encysted zoospores
   C. Zoospores with flagella
   D. Secreted products
   E. Flagella part
2. Which species has been reported to cause vascular pythiosis in only a few cases? A. *Pythium ultimum*
   B. *Pythium irregulare*
   C. *Pythium insidiosum*
   D. *Pythium aphanidermatum*
   E. *Phytophthora spp.*

**Quiz 3 (n=3)**

1. Which specimen type and transport method are most appropriate if transportation takes more than 4 hours to diagnose vascular pythiosis?

A. Send arterial wall in a sterile container without freezing
B. Send thrombus in a sterile container with freezing
C. Send whole blood in a sterile blood collection tube with freezing
D. Send plasma/serum in a sterile blood collection tube with freezing
E. Send skin sample from the lesion site in a sterile container without freezing

1. Which patient specimen is most likely to yield a positive culture result in vascular pythiosis?

A. Arterial wall
B. Thrombus in the artery
C. Blood sample
D. Tissue sample from lesion site
E. Skin from lesion site

1. What is the most logical next step for a patient suspected of vascular pythiosis with negative culture but positive beta-D-glucan test?

A. Rule out Pythiosis due to negative culture
B. Suspect other fungal infection due to positive beta-D-glucan
C. Test for *Pythium insidiosum* antibodies
D. Repeat blood culture
E. Perform KOH prep on remaining tissue for rare-septate hyphae

**Quiz 4 (n=3)**

1. For chronic kidney disease patients with vascular pythiosis, which imaging method is most appropriate to assess disease severity?

A. Plain radiograph
B. Plain MDCT scan
C. PET/CT scan
D. Angiogram using CO2 as contrast agent
E. Non-contrast enhanced MRA

1. A 45-year-old woman presents with worsening left thigh pain, swelling, and cold, pale left leg. CTA of lower extremities is performed. Which radiologic finding is expected? (4 choices)

A. Arterial occlusion
B. Aneurysm
C. Pseudoaneurysm
D. Arteritis

1. Vascular pythiosis is associated with thalassemia, commonly seen in Thailand. From the CT scan, which finding is NOT expected in this patient? (4 choices)


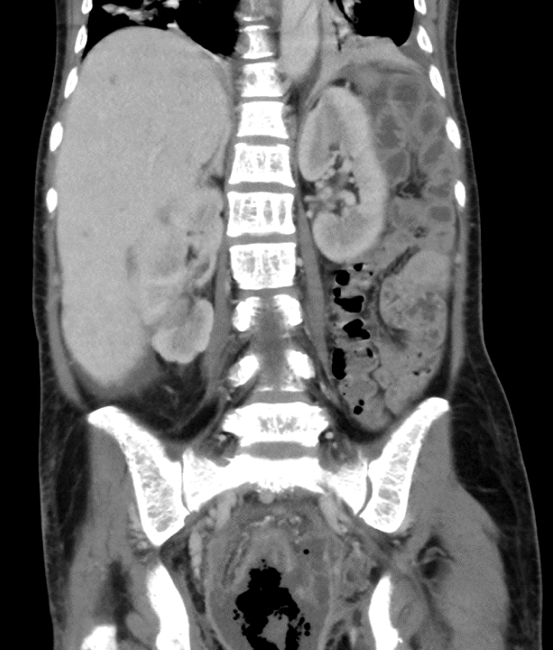


A. Hepatomegaly
B. Post-splenectomy changes
C. Coarse trabeculation of bones
D. Paraspinal extramedullary hematopoiesis

**Quiz 5 (n=3)**

1. What is the most effective treatment to improve survival in vascular pythiosis?

A. Antifungal medication
B. Antibacterial medication
C. Antiviral medication
D. Surgical removal of infected tissue
E. Vaccination

1. Which drug combination has shown synergistic effects against *P. insidiosum*?

A. Voriconazole + Micafungin
B. Amphotericin + Micafungin
C. Azithromycin + Doxycycline
D. Itraconazole + Terbinafine
E. Voriconazole + Terbinafine

1. A 40-year-old male diagnosed with common iliac artery pythiosis showed improvement with decreased beta-D-glucan and wound healing. How long should antifungal treatment continue?

A. 1 month
B. 3 months
C. 6 months
D. 12 months
E. 18 months

**Quiz 6 (n=3)**

1. Which medication is known to cause photosensitivity as a side effect?

A. Itraconazole
B. Terbinafine
C. Levofloxacin
D. Doxycycline
E. Azithromycin

1. Which antibacterial drug class can be used to treat vascular pythiosis?

A. β-lactam
B. Aminoglycoside
C. Glycopeptide
D. Fluoroquinolone
E. Macrolide

1. A 60-year-old Thai male receiving treatment for vascular pythiosis with itraconazole and antibacterial drugs has an itraconazole level of 0.1 mg/L (optimal level is 1 mg/L). How should treatment be adjusted?

A. Take medication 1 hour before meals
B. Take medication with soda or acidic drinks
C. Take medication with antacids
D. Increase dose to 400 mg twice daily
E. Add probenecid

**Quiz 7 (n=3)**

1. Which of the following statements is true regarding vascular pythiosis and surgical treatment?

A. Surgery does not need to achieve clear margins
B. *Pythium insidiosum* is a type of fungus
C. Patients with vascular pythiosis in the leg can be treated with medication alone if the wound starts healing
D. Iliac artery pythiosis cannot be surgically treated
E. Vascular pythiosis can present with both acute and chronic arterial occlusion

1. Which of the following is NOT a symptom of vascular pythiosis?

A. Chronic leg ulcer
B. Walking claudication
C. Acute leg pain
D. Arterial aneurysm
E. Peripheral neuropathy

1. A 45-year-old male diagnosed with vascular Pythiosis shows arterial occlusion of the left superficial femoral artery, popliteal artery, and tibial artery on CTA. While waiting for *Pythium* antibody results, he develops worsening left leg pain and gangrene. What is the appropriate management?

A. Left above-knee amputation
B. Left knee disarticulation
C. Open biopsy of the left popliteal artery for pathology and KOH prep
D. Test ESR and CRP levels
E. Administer heparin and painkillers

**Table A. Quiz scores after each lesson categorized by educational levels**

| Group | Quiz 1  (3 points) | Quiz 2  (3 points) | Quiz 3  (3 points) | Quiz 4  (3 points) | Quiz 5  (3 points) | Quiz 6  (3 points) | Quiz 7  (3 points) | Total  (21 points) |
| --- | --- | --- | --- | --- | --- | --- | --- | --- |
| <BC |  |  |  |  |  |  |  |  |
| Mean | 2.86 | 2.69 | 2.81 | 2.80 | 2.77 | 2.75 | 2.82 | 19.62 |
| Median | 3 | 3 | 3 | 3 | 3 | 3 | 3 | 21 |
| BC |  |  |  |  |  |  |  |  |
| Mean | 2.90 | 2.72 | 2.93 | 2.73 | 2.75 | 2.79 | 2.82 | 19.63 |
| Median | 3 | 3 | 3 | 3 | 3 | 3 | 3 | 21 |
| >BC |  |  |  |  |  |  |  |  |
| Mean | 2.92 | 2.60 | 2.80 | 2.88 | 2.88 | 2.72 | 2.80 | 19.44 |
| Median | 3 | 3 | 3 | 3 | 3 | 3 | 3 | 21 |
