# Supplementary material for: Enhancing knowledge of vascular pythiosis: Impact of a self-paced online course among Thai learners
Source: PLoS Negl Trop Dis. 2025 Apr 8;19(4):e0013003. doi: 10.1371/journal.pntd.0013003 (PMC12005490; doi:10.1371/journal.pntd.0013003)
Supplement: S2 File — (PDF) [file pntd.0013003.s002.pdf]

| student ID | group | pretest | Quiz1 | Quiz2 | Quiz3 | Quiz4 | Quiz5 | Quiz6 | Quiz7 | posttest |
|---|---|---|---|---|---|---|---|---|---|---|
| 2 | 3 | 7 | 3 | 3 | 3 | 3 | 3 | 3 | 3 | 7 |
| 6 | 3 | 5 | 3 | 3 | 3 | 3 | 3 | 3 | 3 | 11 |
| 8 | 1 | 12 | 3 | 3 | 3 | 3 | 3 | 3 | 3 | 12 |
| 10 | 1 | 7 | 3 | 3 | 3 | 3 | 3 | 3 | 3 | 10 |
| 14 | 1 | 4 | 3 | 3 | 3 | 3 | 3 | 3 | 3 | 11 |
| 19 | 1 | 3 | 3 | 3 | 3 | 3 | 3 | 3 | 3 | 9 |
| 20 | 2 | 2 | 3 | 3 | 3 | 3 | 3 | 3 | 3 | 12 |
| 23 | 1 | 4 | 3 | 3 | 3 | 3 | 3 | 3 | 3 | 8 |
| 26 | 2 | 3 | 3 | 3 | 3 | 3 | 3 | 3 | 3 | 3 |
| 29 | 2 | 3 | 3 | 3 | 3 | 3 | 3 | 3 | 2 | 7 |
| 30 | 1 | 1 | 3 | 3 | 3 | 3 | 3 | 3 | 3 | 9 |
| 31 | 1 | 3 | 2 | 1 | 2 | 3 | 2 | 1 | 2 | 9 |
| 35 | 2 | 2 | 3 | 3 | 3 | 3 | 3 | 3 | 3 | 10 |
| 36 | 2 | 4 | 3 | 3 | 3 | 3 | 3 | 3 | 3 | 9 |
| 39 | 2 | 3 | 3 | 3 | 3 | 3 | 2 | 3 | 2 | 6 |
| 40 | 3 | 4 | 3 | 3 | 3 | 3 | 3 | 3 | 3 | 9 |
| 42 | 2 | 3 | 3 | 3 | 3 | 3 | 3 | 3 | 3 | 11 |
| 49 | 1 | 6 | 3 | 3 | 3 | 3 | 3 | 3 | 3 | 11 |
| 50 | 3 | 11 | 3 | 3 | 3 | 3 | 3 | 3 | 3 | 12 |
| 52 | 1 | 5 | 3 | 3 | 3 | 3 | 3 | 3 | 3 | 9 |
| 54 | 1 | 2 | 3 | 3 | 3 | 3 | 3 | 3 | 3 | 9 |
| 61 | 1 | 3 | 3 | 3 | 3 | 3 | 3 | 3 | 3 | 5 |
| 66 | 2 | 12 | 3 | 3 | 3 | 3 | 3 | 3 | 3 | 12 |
| 70 | 3 | 9 | 3 | 3 | 3 | 3 | 3 | 3 | 3 | 11 |
| 72 | 2 | 3 | 3 | 3 | 3 | 3 | 3 | 3 | 3 | 9 |
| 73 | 2 | 3 | 3 | 3 | 3 | 3 | 3 | 3 | 3 | 9 |
| 79 | 1 | 12 | 3 | 3 | 3 | 3 | 3 | 3 | 3 | 12 |
| 80 | 1 | 12 | 3 | 3 | 3 | 3 | 3 | 3 | 3 | 12 |
| 81 | 2 | 6 | 3 | 3 | 3 | 3 | 3 | 3 | 1 | 6 |
| 83 | 1 | 12 | 3 | 3 | 3 | 3 | 3 | 3 | 3 | 12 |
| 100 | 2 | 1 | 3 | 0 | 1 | 1 | 3 | 3 | 2 | 7 |
| 107 | 1 | 1 | 3 | 3 | 3 | 3 | 3 | 3 | 3 | 9 |
| 119 | 2 | 5 | 3 | 3 | 3 | 3 | 3 | 3 | 3 | 9 |
| 128 | 3 | 2 | 3 | 3 | 3 | 3 | 3 | 3 | 3 | 7 |
| 134 | 1 | 0 | 3 | 3 | 3 | 3 | 3 | 3 | 3 | 9 |
| 141 | 2 | 2 | 3 | 3 | 3 | 3 | 3 | 3 | 3 | 6 |
| 145 | 1 | 2 | 3 | 3 | 3 | 3 | 3 | 3 | 3 | 7 |
| 148 | 2 | 6 | 3 | 3 | 3 | 3 | 3 | 3 | 3 | 6 |
| 149 | 3 | 12 | 3 | 3 | 3 | 3 | 3 | 3 | 3 | 12 |
| 150 | 1 | 6 | 3 | 3 | 3 | 3 | 3 | 3 | 3 | 10 |
| 152 | 1 | 7 | 3 | 3 | 3 | 3 | 3 | 3 | 3 | 8 |
| 157 | 1 | 12 | 3 | 3 | 3 | 3 | 3 | 3 | 3 | 12 |
| 164 | 2 | 12 | 3 | 3 | 3 | 3 | 3 | 3 | 3 | 12 |
| 169 | 1 | 1 | 3 | 3 | 3 | 3 | 3 | 3 | 3 | 4 |
| 178 | 2 | 6 | 3 | 3 | 3 | 3 | 3 | 3 | 3 | 6 |
| 179 | 2 | 7 | 3 | 3 | 3 | 3 | 3 | 3 | 3 | 6 |
| 185 | 2 | 1 | 3 | 3 | 3 | 3 | 3 | 3 | 3 | 7 |

| | | | | | | | | | | |
|---|---|---|---|---|---|---|---|---|---|---|
| 194 | 2 | 4 | 3 | 3 | 3 | 3 | 3 | 3 | 3 | 7 |
| 207 | 2 | 5 | 3 | 1 | 3 | 1 | 3 | 3 | 3 | 7 |
| 210 | 2 | 1 | 3 | 3 | 3 | 3 | 3 | 3 | 3 | 5 |
| 218 | 1 | 6 | 3 | 3 | 3 | 3 | 3 | 3 | 3 | 7 |
| 221 | 1 | 2 | 3 | 3 | 3 | 3 | 3 | 3 | 3 | 7 |
| 222 | 1 | 4 | 3 | 2 | 2 | 3 | 3 | 3 | 3 | 8 |
| 224 | 3 | 7 | 3 | 3 | 3 | 3 | 3 | 3 | 3 | 7 |
| 227 | 1 | 5 | 3 | 3 | 3 | 3 | 3 | 3 | 3 | 5 |
| 230 | 1 | 2 | 3 | 3 | 3 | 3 | 3 | 3 | 3 | 3 |
| 231 | 1 | 4 | 3 | 3 | 3 | 3 | 3 | 3 | 3 | 9 |
| 233 | 2 | 2 | 3 | 3 | 3 | 3 | 3 | 3 | 3 | 8 |
| 238 | 2 | 1 | 3 | 3 | 3 | 3 | 3 | 3 | 3 | 7 |
| 243 | 2 | 3 | 2 | 3 | 3 | 3 | 3 | 3 | 3 | 4 |
| 246 | 1 | 5 | 3 | 3 | 3 | 3 | 3 | 3 | 3 | 6 |
| 252 | 1 | 3 | 3 | 3 | 3 | 3 | 3 | 3 | 3 | 8 |
| 260 | 1 | 3 | 3 | 3 | 3 | 3 | 3 | 3 | 3 | 3 |
| 263 | 1 | 4 | 3 | 2 | 3 | 2 | 3 | 3 | 3 | 8 |
| 286 | 1 | 4 | 3 | 2 | 3 | 3 | 2 | 0 | 0 | 5 |
| 289 | 1 | 2 | 3 | 3 | 3 | 3 | 3 | 3 | 3 | 4 |
| 294 | 1 | 4 | 3 | 3 | 3 | 3 | 3 | 3 | 3 | 5 |
| 298 | 2 | 2 | 3 | 3 | 3 | 3 | 3 | 3 | 3 | 7 |
| 299 | 2 | 3 | 3 | 3 | 3 | 3 | 3 | 3 | 3 | 7 |
| 302 | 1 | 8 | 3 | 3 | 3 | 3 | 3 | 3 | 3 | 7 |
| 304 | 1 | 1 | 3 | 3 | 3 | 3 | 3 | 3 | 3 | 6 |
| 306 | 1 | 3 | 3 | 3 | 3 | 3 | 3 | 3 | 3 | 7 |
| 310 | 1 | 3 | 3 | 3 | 3 | 3 | 3 | 3 | 3 | 2 |
| 313 | 1 | 4 | 2 | 2 | 2 | 3 | 3 | 3 | 3 | 6 |
| 315 | 1 | 3 | 3 | 3 | 3 | 3 | 3 | 3 | 3 | 9 |
| 324 | 1 | 10 | 3 | 3 | 3 | 3 | 3 | 3 | 3 | 11 |
| 330 | 1 | 2 | 3 | 1 | 3 | 3 | 3 | 3 | 3 | 5 |
| 332 | 1 | 4 | 3 | 3 | 3 | 3 | 3 | 3 | 3 | 6 |
| 347 | 3 | 6 | 3 | 3 | 3 | 3 | 3 | 3 | 3 | 11 |
| 348 | 1 | 3 | 3 | 3 | 3 | 3 | 3 | 3 | 3 | 5 |
| 353 | 1 | 2 | 1 | 1 | 1 | 1 | 2 | 1 | 0 | 1 |
| 358 | 1 | 2 | 3 | 3 | 3 | 3 | 3 | 3 | 3 | 7 |
| 362 | 1 | 3 | 3 | 3 | 3 | 3 | 3 | 3 | 3 | 9 |
| 365 | 1 | 1 | 3 | 3 | 3 | 3 | 3 | 3 | 3 | 5 |
| 368 | 2 | 12 | 3 | 3 | 3 | 3 | 3 | 3 | 3 | 12 |
| 369 | 1 | 3 | 3 | 3 | 3 | 3 | 3 | 3 | 3 | 9 |
| 373 | 1 | 4 | 3 | 0 | 3 | 0 | 2 | 0 | 0 | 4 |
| 379 | 1 | 2 | 1 | 3 | 1 | 3 | 3 | 3 | 3 | 2 |
| 380 | 1 | 5 | 3 | 3 | 3 | 3 | 3 | 3 | 3 | 4 |
| 385 | 1 | 1 | 3 | 3 | 3 | 3 | 3 | 3 | 3 | 4 |
| 388 | 1 | 6 | 3 | 3 | 3 | 3 | 3 | 3 | 3 | 8 |
| 395 | 1 | 2 | 3 | 3 | 3 | 3 | 3 | 3 | 3 | 4 |
| 402 | 1 | 3 | 3 | 3 | 3 | 3 | 3 | 3 | 3 | 4 |
| 403 | 1 | 1 | 3 | 3 | 3 | 3 | 3 | 3 | 3 | 10 |
| 404 | 2 | 3 | 3 | 3 | 3 | 2 | 3 | 3 | 3 | 8 |
| 409 | 1 | 1 | 3 | 3 | 3 | 3 | 3 | 3 | 3 | 6 |
| 412 | 1 | 1 | 3 | 3 | 3 | 3 | 3 | 3 | 3 | 7 |

| | | | | | | | | | | |
|---|---|---|---|---|---|---|---|---|---|---|
| 413 | 1 | 1 | 3 | 3 | 3 | 3 | 3 | 3 | 3 | 6 |
| 420 | 1 | 4 | 3 | 3 | 3 | 3 | 3 | 3 | 3 | 1 |
| 423 | 1 | 1 | 3 | 3 | 3 | 3 | 3 | 3 | 3 | 8 |
| 426 | 3 | 1 | 3 | 2 | 3 | 3 | 3 | 3 | 3 | 10 |
| 432 | 1 | 4 | 3 | 3 | 3 | 3 | 3 | 3 | 3 | 8 |
| 441 | 1 | 2 | 1 | 2 | 0 | 0 | 1 | 0 | 2 | 4 |
| 443 | 1 | 2 | 3 | 3 | 3 | 3 | 3 | 3 | 3 | 7 |
| 444 | 2 | 4 | 3 | 0 | 3 | 0 | 1 | 1 | 3 | 4 |
| 458 | 1 | 5 | 3 | 3 | 3 | 3 | 3 | 3 | 3 | 5 |
| 465 | 2 | 9 | 3 | 3 | 3 | 3 | 3 | 3 | 3 | 10 |
| 476 | 1 | 1 | 3 | 3 | 3 | 3 | 3 | 3 | 3 | 8 |
| 480 | 1 | 2 | 3 | 3 | 3 | 3 | 3 | 3 | 3 | 5 |
| 485 | 1 | 2 | 3 | 3 | 3 | 3 | 3 | 3 | 3 | 0 |
| 486 | 1 | 6 | 3 | 3 | 3 | 3 | 3 | 3 | 3 | 6 |
| 487 | 1 | 2 | 3 | 3 | 3 | 3 | 3 | 3 | 3 | 2 |
| 493 | 2 | 6 | 3 | 3 | 3 | 3 | 3 | 3 | 3 | 8 |
| 494 | 1 | 2 | 2 | 3 | 3 | 3 | 3 | 3 | 3 | 4 |
| 500 | 1 | 1 | 3 | 3 | 3 | 3 | 3 | 3 | 3 | 7 |
| 501 | 1 | 3 | 3 | 3 | 3 | 3 | 3 | 3 | 3 | 6 |
| 505 | 1 | 0 | 3 | 1 | 2 | 2 | 3 | 1 | 2 | 7 |
| 506 | 1 | 5 | 3 | 3 | 3 | 3 | 3 | 3 | 3 | 7 |
| 507 | 2 | 5 | 3 | 3 | 3 | 3 | 3 | 3 | 3 | 9 |
| 508 | 1 | 2 | 3 | 3 | 3 | 3 | 3 | 3 | 3 | 12 |
| 511 | 1 | 1 | 2 | 1 | 2 | 2 | 3 | 3 | 3 | 6 |
| 512 | 1 | 7 | 3 | 3 | 3 | 3 | 3 | 3 | 3 | 8 |
| 514 | 2 | 2 | 3 | 3 | 3 | 3 | 3 | 3 | 3 | 5 |
| 516 | 1 | 4 | 3 | 3 | 3 | 3 | 3 | 3 | 3 | 1 |
| 521 | 1 | 3 | 3 | 3 | 3 | 3 | 3 | 3 | 3 | 7 |
| 522 | 1 | 2 | 3 | 3 | 3 | 3 | 3 | 2 | 3 | 6 |
| 524 | 1 | 2 | 3 | 3 | 3 | 3 | 3 | 3 | 3 | 10 |
| 526 | 1 | 6 | 3 | 3 | 3 | 3 | 3 | 3 | 3 | 9 |
| 528 | 1 | 7 | 3 | 3 | 3 | 3 | 3 | 3 | 3 | 7 |
| 530 | 1 | 1 | 3 | 3 | 3 | 3 | 3 | 3 | 3 | 6 |
| 531 | 1 | 0 | 2 | 1 | 2 | 3 | 3 | 1 | 2 | 9 |
| 539 | 2 | 0 | 3 | 1 | 3 | 1 | 2 | 2 | 2 | 7 |
| 540 | 1 | 4 | 1 | 1 | 0 | 0 | 0 | 0 | 1 | 1 |
| 544 | 1 | 7 | 3 | 3 | 3 | 3 | 3 | 3 | 3 | 8 |
| 547 | 1 | 1 | 3 | 3 | 3 | 3 | 3 | 3 | 3 | 8 |
| 556 | 3 | 9 | 3 | 2 | 3 | 3 | 3 | 2 | 3 | 12 |
| 557 | 1 | 2 | 2 | 3 | 3 | 3 | 3 | 3 | 3 | 3 |
| 558 | 1 | 5 | 3 | 3 | 3 | 3 | 3 | 3 | 3 | 9 |
| 559 | 2 | 7 | 3 | 2 | 3 | 3 | 3 | 1 | 2 | 10 |
| 561 | 3 | 11 | 3 | 3 | 3 | 3 | 3 | 3 | 3 | 11 |
| 562 | 1 | 1 | 2 | 3 | 3 | 3 | 3 | 3 | 3 | 6 |
| 563 | 1 | 3 | 3 | 0 | 0 | 1 | 2 | 0 | 3 | 4 |
| 569 | 3 | 5 | 3 | 3 | 3 | 3 | 3 | 3 | 3 | 11 |
| 570 | 3 | 8 | 2 | 2 | 3 | 3 | 2 | 3 | 2 | 11 |
| 576 | 3 | 5 | 3 | 3 | 3 | 3 | 3 | 3 | 3 | 7 |
| 577 | 1 | 2 | 3 | 3 | 3 | 3 | 3 | 3 | 3 | 6 |
| 578 | 1 | 3 | 3 | 3 | 2 | 3 | 3 | 3 | 3 | 10 |

| | | | | | | | | | |
|---|---|---|---|---|---|---|---|---|---|
| 581 | 2 | 4 | 3 | 3 | 3 | 3 | 3 | 3 | 3 | 9 |
| 584 | 1 | 2 | 2 | 2 | 2 | 1 | 2 | 3 | 3 | 7 |
| 587 | 3 | 1 | 3 | 3 | 3 | 3 | 3 | 3 | 3 | 3 |
| 588 | 1 | 1 | 2 | 3 | 3 | 2 | 2 | 3 | 3 | 4 |
| 591 | 2 | 5 | 3 | 2 | 3 | 3 | 3 | 3 | 3 | 9 |
| 596 | 1 | 5 | 3 | 3 | 3 | 3 | 3 | 3 | 3 | 8 |
| 601 | 1 | 7 | 3 | 3 | 3 | 3 | 3 | 3 | 3 | 7 |
| 602 | 3 | 3 | 3 | 1 | 3 | 3 | 3 | 3 | 3 | 10 |
| 604 | 1 | 1 | 3 | 3 | 3 | 3 | 3 | 3 | 3 | 8 |
| 606 | 1 | 2 | 3 | 3 | 3 | 3 | 3 | 3 | 3 | 10 |
| 615 | 1 | 0 | 3 | 3 | 3 | 3 | 3 | 3 | 3 | 9 |
| 630 | 2 | 2 | 3 | 3 | 3 | 3 | 3 | 3 | 3 | 1 |
| 639 | 1 | 2 | 3 | 3 | 3 | 3 | 3 | 3 | 3 | 8 |
| 642 | 1 | 5 | 3 | 3 | 3 | 2 | 2 | 2 | 3 | 8 |
| 643 | 1 | 4 | 2 | 1 | 2 | 2 | 3 | 3 | 2 | 6 |
| 646 | 1 | 3 | 3 | 3 | 3 | 3 | 3 | 3 | 3 | 8 |
| 647 | 1 | 4 | 3 | 3 | 3 | 3 | 3 | 3 | 3 | 7 |
| 653 | 1 | 7 | 3 | 3 | 3 | 3 | 3 | 3 | 3 | 8 |
| 655 | 1 | 6 | 3 | 3 | 3 | 3 | 2 | 3 | 3 | 6 |
| 658 | 1 | 1 | 3 | 3 | 3 | 1 | 3 | 2 | 3 | 8 |
| 660 | 1 | 9 | 3 | 3 | 3 | 3 | 3 | 3 | 3 | 9 |
| 666 | 1 | 4 | 3 | 3 | 3 | 3 | 3 | 3 | 3 | 3 |
| 668 | 1 | 5 | 3 | 3 | 2 | 3 | 3 | 3 | 3 | 7 |
| 672 | 1 | 2 | 3 | 3 | 3 | 3 | 3 | 3 | 3 | 9 |
| 673 | 1 | 4 | 3 | 3 | 3 | 3 | 3 | 3 | 3 | 8 |
| 681 | 1 | 1 | 2 | 0 | 1 | 1 | 1 | 0 | 1 | 3 |
| 683 | 1 | 6 | 3 | 3 | 3 | 3 | 3 | 3 | 3 | 10 |
| 684 | 1 | 2 | 3 | 3 | 3 | 3 | 3 | 3 | 3 | 3 |
| 687 | 3 | 10 | 3 | 2 | 3 | 3 | 2 | 3 | 2 | 10 |
| 691 | 1 | 2 | 3 | 0 | 3 | 3 | 3 | 3 | 2 | 3 |
| 696 | 1 | 5 | 3 | 3 | 3 | 3 | 3 | 3 | 3 | 10 |
| 697 | 1 | 3 | 3 | 3 | 3 | 3 | 3 | 3 | 3 | 8 |
| 699 | 1 | 3 | 2 | 0 | 2 | 2 | 3 | 3 | 3 | 7 |
| 701 | 2 | 7 | 3 | 3 | 3 | 3 | 3 | 3 | 3 | 9 |
| 705 | 1 | 3 | 3 | 1 | 2 | 2 | 3 | 0 | 1 | 7 |
| 711 | 1 | 2 | 3 | 3 | 3 | 3 | 3 | 3 | 3 | 2 |
| 715 | 2 | 2 | 3 | 3 | 3 | 3 | 3 | 3 | 3 | 3 |
| 724 | 1 | 3 | 3 | 3 | 3 | 3 | 3 | 3 | 2 | 3 |
| 734 | 1 | 3 | 3 | 3 | 3 | 3 | 3 | 3 | 3 | 5 |
| 740 | 1 | 7 | 3 | 3 | 3 | 3 | 3 | 3 | 3 | 7 |
| 742 | 1 | 2 | 3 | 3 | 3 | 3 | 3 | 3 | 3 | 10 |
| 748 | 1 | 2 | 3 | 3 | 3 | 3 | 3 | 3 | 3 | 5 |
| 755 | 1 | 4 | 3 | 3 | 2 | 3 | 3 | 2 | 3 | 4 |
| 761 | 1 | 4 | 3 | 2 | 3 | 3 | 3 | 2 | 3 | 5 |
| 762 | 1 | 6 | 2 | 2 | 2 | 2 | 3 | 2 | 2 | 8 |
| 763 | 1 | 4 | 3 | 3 | 3 | 3 | 3 | 3 | 3 | 8 |
| 773 | 1 | 1 | 3 | 3 | 3 | 3 | 3 | 3 | 3 | 4 |
| 774 | 1 | 3 | 3 | 3 | 3 | 3 | 3 | 3 | 3 | 5 |
| 778 | 1 | 2 | 3 | 3 | 3 | 3 | 3 | 3 | 3 | 7 |
| 780 | 1 | 4 | 2 | 3 | 3 | 3 | 3 | 3 | 3 | 4 |

| | | | | | | | | | | |
|---|---|---|---|---|---|---|---|---|---|---|
| 781 | 1 | 0 | 3 | 3 | 3 | 3 | 3 | 3 | 3 | 10 |
| 788 | 1 | 3 | 3 | 3 | 3 | 3 | 3 | 3 | 3 | 6 |
| 797 | 1 | 3 | 3 | 3 | 3 | 3 | 3 | 0 | 3 | 6 |
| 804 | 1 | 4 | 3 | 3 | 3 | 3 | 3 | 3 | 3 | 2 |
| 806 | 1 | 3 | 3 | 3 | 3 | 3 | 3 | 3 | 3 | 8 |
| 810 | 1 | 5 | 3 | 3 | 2 | 1 | 3 | 3 | 2 | 6 |
| 812 | 1 | 4 | 3 | 3 | 3 | 3 | 3 | 3 | 3 | 5 |
| 814 | 1 | 3 | 3 | 3 | 3 | 3 | 3 | 3 | 3 | 9 |
| 818 | 1 | 4 | 3 | 3 | 3 | 3 | 3 | 3 | 3 | 7 |
| 819 | 1 | 1 | 3 | 3 | 3 | 3 | 3 | 3 | 3 | 6 |
| 820 | 1 | 0 | 3 | 3 | 3 | 3 | 3 | 3 | 3 | 10 |
| 827 | 2 | 3 | 3 | 3 | 3 | 3 | 3 | 3 | 3 | 4 |
| 829 | 1 | 2 | 2 | 1 | 2 | 2 | 3 | 1 | 1 | 8 |
| 837 | 2 | 2 | 3 | 3 | 3 | 3 | 3 | 3 | 3 | 8 |
| 838 | 1 | 7 | 3 | 3 | 3 | 3 | 3 | 3 | 3 | 8 |
| 844 | 1 | 5 | 3 | 3 | 3 | 3 | 3 | 3 | 2 | 3 |
| 850 | 1 | 5 | 2 | 1 | 1 | 2 | 2 | 0 | 2 | 9 |
| 851 | 1 | 0 | 3 | 1 | 2 | 2 | 2 | 0 | 0 | 3 |
| 863 | 1 | 5 | 3 | 3 | 3 | 1 | 3 | 2 | 2 | 10 |
| 869 | 1 | 2 | 3 | 3 | 3 | 3 | 3 | 3 | 3 | 5 |
| 873 | 1 | 2 | 3 | 3 | 3 | 3 | 3 | 3 | 3 | 9 |
| 875 | 1 | 4 | 2 | 1 | 0 | 1 | 1 | 1 | 1 | 6 |
| 876 | 1 | 4 | 2 | 3 | 3 | 3 | 3 | 3 | 3 | 8 |
| 881 | 1 | 4 | 3 | 3 | 3 | 3 | 3 | 3 | 3 | 8 |
| 885 | 1 | 6 | 3 | 3 | 3 | 3 | 3 | 3 | 3 | 3 |
| 890 | 2 | 4 | 3 | 3 | 3 | 3 | 3 | 3 | 3 | 8 |
| 893 | 1 | 4 | 3 | 3 | 3 | 3 | 3 | 3 | 3 | 5 |
| 909 | 1 | 4 | 3 | 3 | 3 | 3 | 3 | 3 | 3 | 8 |
| 912 | 1 | 4 | 3 | 3 | 3 | 3 | 3 | 3 | 3 | 7 |
| 913 | 1 | 2 | 3 | 3 | 3 | 3 | 3 | 3 | 3 | 6 |
| 925 | 1 | 2 | 3 | 3 | 3 | 3 | 3 | 3 | 3 | 9 |
| 927 | 1 | 4 | 3 | 3 | 3 | 3 | 3 | 3 | 3 | 2 |
| 928 | 1 | 2 | 3 | 3 | 3 | 3 | 3 | 3 | 3 | 7 |
| 930 | 1 | 5 | 3 | 3 | 3 | 3 | 3 | 3 | 3 | 1 |
| 932 | 1 | 1 | 2 | 3 | 3 | 3 | 3 | 3 | 3 | 7 |
| 933 | 1 | 2 | 2 | 3 | 1 | 2 | 2 | 1 | 3 | 4 |
| 934 | 1 | 1 | 3 | 3 | 3 | 3 | 3 | 3 | 3 | 8 |
| 935 | 2 | 1 | 2 | 3 | 3 | 3 | 3 | 3 | 3 | 3 |
| 936 | 1 | 6 | 3 | 3 | 3 | 3 | 3 | 3 | 3 | 5 |
| 940 | 1 | 3 | 3 | 3 | 3 | 3 | 3 | 3 | 3 | 6 |
| 941 | 1 | 2 | 3 | 3 | 3 | 3 | 3 | 3 | 3 | 8 |
| 945 | 1 | 3 | 3 | 3 | 3 | 3 | 3 | 3 | 3 | 6 |
| 950 | 1 | 4 | 3 | 3 | 3 | 3 | 3 | 3 | 3 | 5 |
| 952 | 1 | 1 | 3 | 3 | 3 | 3 | 3 | 3 | 3 | 9 |
| 958 | 1 | 2 | 3 | 2 | 3 | 3 | 3 | 3 | 2 | 7 |
| 960 | 1 | 4 | 3 | 3 | 3 | 3 | 3 | 3 | 1 | 8 |
| 966 | 1 | 4 | 3 | 3 | 3 | 3 | 3 | 3 | 3 | 9 |
| 969 | 1 | 3 | 3 | 0 | 2 | 2 | 2 | 0 | 3 | 8 |
| 971 | 1 | 6 | 3 | 3 | 3 | 3 | 3 | 3 | 3 | 8 |
| 976 | 1 | 3 | 3 | 3 | 3 | 3 | 3 | 3 | 3 | 4 |

| | | | | | | | | | | |
|---|---|---|---|---|---|---|---|---|---|---|
| 978 | 2 | 3 | 3 | 3 | 3 | 3 | 3 | 3 | 3 | 9 |
| 980 | 1 | 5 | 3 | 3 | 3 | 3 | 3 | 3 | 3 | 8 |
| 982 | 1 | 2 | 2 | 3 | 3 | 3 | 3 | 3 | 3 | 5 |
| 984 | 1 | 1 | 3 | 3 | 3 | 3 | 3 | 3 | 3 | 3 |
| 986 | 1 | 1 | 3 | 3 | 3 | 3 | 3 | 3 | 3 | 1 |
| 989 | 1 | 0 | 3 | 3 | 3 | 3 | 3 | 3 | 3 | 6 |
| 993 | 1 | 2 | 3 | 3 | 3 | 3 | 3 | 3 | 3 | 0 |
| 994 | 1 | 1 | 3 | 3 | 3 | 3 | 3 | 3 | 3 | 2 |
| 995 | 1 | 2 | 3 | 3 | 3 | 3 | 3 | 3 | 3 | 9 |
| 997 | 1 | 1 | 3 | 3 | 3 | 3 | 3 | 3 | 3 | 4 |
| 1007 | 1 | 1 | 3 | 3 | 3 | 3 | 3 | 3 | 3 | 1 |
| 1008 | 1 | 5 | 3 | 3 | 3 | 3 | 3 | 3 | 3 | 3 |
| 1016 | 1 | 4 | 3 | 3 | 2 | 3 | 3 | 3 | 3 | 8 |
| 1019 | 1 | 6 | 3 | 3 | 3 | 3 | 3 | 3 | 3 | 3 |
| 1020 | 1 | 5 | 3 | 3 | 3 | 3 | 3 | 3 | 3 | 2 |
| 1022 | 1 | 5 | 3 | 3 | 3 | 3 | 3 | 3 | 3 | 8 |
| 1029 | 3 | 9 | 3 | 3 | 3 | 3 | 3 | 3 | 3 | 9 |
| 1038 | 1 | 3 | 3 | 2 | 3 | 3 | 3 | 2 | 3 | 6 |
| 1047 | 1 | 2 | 3 | 1 | 3 | 3 | 3 | 3 | 3 | 7 |
| 1048 | 1 | 1 | 3 | 3 | 3 | 3 | 3 | 3 | 3 | 8 |
| 1049 | 1 | 1 | 3 | 1 | 3 | 2 | 3 | 2 | 3 | 8 |
| 1050 | 1 | 4 | 3 | 3 | 3 | 2 | 3 | 3 | 3 | 11 |
| 1051 | 1 | 5 | 3 | 3 | 3 | 3 | 3 | 3 | 3 | 7 |
| 1057 | 1 | 2 | 3 | 3 | 3 | 3 | 3 | 3 | 3 | 6 |
| 1064 | 1 | 7 | 3 | 3 | 3 | 3 | 3 | 3 | 3 | 6 |
| 1066 | 1 | 0 | 3 | 3 | 3 | 3 | 3 | 3 | 3 | 5 |
| 1068 | 1 | 6 | 3 | 3 | 3 | 3 | 3 | 3 | 3 | 7 |
| 1069 | 1 | 0 | 3 | 3 | 3 | 3 | 3 | 3 | 3 | 5 |
| 1073 | 1 | 2 | 3 | 2 | 3 | 2 | 3 | 2 | 2 | 6 |
| 1075 | 1 | 4 | 3 | 3 | 3 | 3 | 3 | 3 | 3 | 7 |
| 1077 | 1 | 2 | 3 | 3 | 3 | 3 | 3 | 3 | 3 | 3 |
| 1079 | 1 | 4 | 3 | 3 | 3 | 3 | 3 | 3 | 3 | 9 |
| 1081 | 1 | 2 | 3 | 3 | 3 | 3 | 3 | 3 | 3 | 8 |
| 1083 | 1 | 7 | 3 | 3 | 3 | 3 | 3 | 3 | 3 | 9 |
| 1084 | 2 | 0 | 3 | 3 | 3 | 3 | 3 | 3 | 3 | 9 |
| 1085 | 1 | 4 | 3 | 3 | 3 | 3 | 3 | 3 | 3 | 8 |
| 1089 | 2 | 5 | 3 | 3 | 3 | 3 | 3 | 3 | 3 | 8 |
| 1090 | 2 | 1 | 3 | 3 | 3 | 3 | 3 | 3 | 3 | 8 |
| 1091 | 3 | 5 | 3 | 3 | 3 | 3 | 3 | 3 | 3 | 10 |
| 1094 | 1 | 1 | 3 | 3 | 3 | 3 | 3 | 3 | 3 | 6 |
| 1096 | 1 | 3 | 3 | 3 | 3 | 3 | 3 | 3 | 3 | 8 |
| 1097 | 3 | 4 | 3 | 3 | 3 | 3 | 3 | 3 | 3 | 10 |
| 1098 | 1 | 4 | 2 | 2 | 2 | 3 | 3 | 3 | 3 | 6 |
| 1099 | 1 | 2 | 3 | 3 | 3 | 3 | 3 | 3 | 3 | 6 |
| 1100 | 1 | 3 | 3 | 2 | 1 | 1 | 2 | 1 | 3 | 5 |
| 1101 | 1 | 0 | 3 | 3 | 3 | 3 | 3 | 3 | 3 | 6 |
| 1102 | 1 | 1 | 3 | 3 | 3 | 2 | 3 | 3 | 3 | 5 |
| 1106 | 1 | 7 | 3 | 3 | 3 | 3 | 3 | 3 | 3 | 5 |
| 1108 | 1 | 0 | 3 | 3 | 3 | 3 | 3 | 3 | 3 | 10 |
| 1109 | 1 | 3 | 3 | 0 | 3 | 3 | 3 | 3 | 3 | 7 |

| | | | | | | | | | | |
|---|---|---|---|---|---|---|---|---|---|---|
| 1110 | 3 | 1 | 3 | 1 | 1 | 2 | 1 | 0 | 3 | 3 |
| 1119 | 1 | 2 | 3 | 3 | 3 | 3 | 3 | 3 | 3 | 3 |
| 1121 | 1 | 3 | 3 | 3 | 3 | 3 | 3 | 3 | 3 | 6 |
| 1122 | 1 | 3 | 3 | 1 | 3 | 1 | 2 | 1 | 2 | 7 |
| 1125 | 1 | 2 | 0 | 1 | 1 | 1 | 1 | 1 | 1 | 1 |
| 1130 | 1 | 3 | 3 | 3 | 3 | 3 | 3 | 3 | 3 | 6 |
| 1131 | 1 | 1 | 2 | 3 | 3 | 3 | 3 | 3 | 3 | 6 |
| 1135 | 1 | 3 | 3 | 3 | 3 | 3 | 3 | 3 | 3 | 1 |
| 1137 | 1 | 1 | 3 | 3 | 3 | 3 | 3 | 3 | 3 | 10 |
| 1141 | 1 | 2 | 3 | 2 | 3 | 2 | 3 | 2 | 2 | 7 |
| 1143 | 1 | 5 | 3 | 3 | 3 | 3 | 3 | 3 | 3 | 9 |
| 1148 | 1 | 9 | 3 | 3 | 3 | 3 | 3 | 3 | 3 | 9 |
| 1150 | 1 | 1 | 3 | 3 | 3 | 3 | 3 | 3 | 3 | 5 |
| 1153 | 1 | 3 | 3 | 3 | 3 | 3 | 3 | 3 | 3 | 4 |
| 1161 | 1 | 2 | 3 | 3 | 3 | 3 | 3 | 3 | 3 | 6 |
| 1164 | 1 | 4 | 3 | 3 | 3 | 2 | 2 | 2 | 2 | 5 |
| 1167 | 1 | 3 | 3 | 3 | 3 | 3 | 3 | 3 | 3 | 1 |
| 1168 | 2 | 8 | 2 | 2 | 3 | 2 | 2 | 3 | 2 | 10 |
| 1173 | 1 | 1 | 3 | 3 | 3 | 3 | 3 | 3 | 3 | 4 |
| 1174 | 1 | 3 | 3 | 3 | 3 | 3 | 3 | 3 | 3 | 7 |
| 1178 | 1 | 1 | 3 | 3 | 3 | 3 | 3 | 3 | 3 | 9 |
| 1179 | 1 | 5 | 3 | 3 | 3 | 3 | 3 | 3 | 3 | 6 |
| 1180 | 1 | 5 | 3 | 0 | 2 | 2 | 2 | 2 | 2 | 5 |
| 1186 | 1 | 3 | 3 | 3 | 2 | 1 | 3 | 1 | 1 | 7 |
| 1187 | 1 | 5 | 3 | 3 | 3 | 3 | 3 | 3 | 3 | 4 |
| 1188 | 1 | 5 | 2 | 2 | 2 | 3 | 3 | 2 | 2 | 9 |
| 1189 | 1 | 2 | 3 | 3 | 3 | 3 | 3 | 3 | 3 | 8 |
| 1190 | 1 | 2 | 3 | 3 | 3 | 3 | 3 | 3 | 3 | 4 |
| 1194 | 1 | 5 | 3 | 3 | 3 | 3 | 3 | 3 | 3 | 6 |
| 1195 | 1 | 2 | 2 | 3 | 3 | 3 | 3 | 3 | 3 | 5 |
| 1199 | 1 | 4 | 3 | 3 | 3 | 3 | 3 | 3 | 3 | 9 |
| 1200 | 1 | 5 | 3 | 2 | 3 | 3 | 3 | 3 | 3 | 7 |
| 1207 | 1 | 2 | 3 | 2 | 3 | 2 | 3 | 3 | 3 | 9 |
| 1209 | 1 | 3 | 3 | 0 | 2 | 1 | 2 | 1 | 1 | 7 |
| 1211 | 1 | 5 | 3 | 3 | 3 | 3 | 3 | 3 | 3 | 5 |
| 1219 | 1 | 4 | 3 | 3 | 3 | 3 | 3 | 3 | 3 | 10 |
| 1225 | 1 | 1 | 3 | 3 | 3 | 3 | 3 | 3 | 3 | 8 |
| 1231 | 2 | 2 | 3 | 3 | 3 | 3 | 3 | 3 | 3 | 5 |
| 1235 | 1 | 3 | 3 | 3 | 3 | 3 | 3 | 3 | 3 | 7 |
| 1238 | 1 | 2 | 3 | 3 | 3 | 3 | 3 | 3 | 3 | 5 |
| 1239 | 1 | 3 | 3 | 3 | 3 | 3 | 3 | 3 | 3 | 9 |
| 1243 | 1 | 4 | 3 | 3 | 3 | 3 | 3 | 3 | 3 | 4 |
| 1245 | 1 | 9 | 3 | 3 | 3 | 3 | 3 | 3 | 3 | 9 |
| 1249 | 1 | 5 | 3 | 1 | 3 | 3 | 3 | 3 | 3 | 9 |
| 1254 | 2 | 4 | 3 | 2 | 3 | 2 | 1 | 1 | 2 | 10 |
| 1256 | 3 | 3 | 2 | 1 | 0 | 1 | 0 | 0 | 0 | 4 |
| 1257 | 1 | 3 | 3 | 3 | 3 | 3 | 3 | 3 | 3 | 10 |
| 1258 | 1 | 3 | 3 | 3 | 3 | 3 | 3 | 3 | 3 | 10 |
| 1261 | 1 | 2 | 3 | 3 | 3 | 3 | 3 | 3 | 3 | 9 |
| 1262 | 1 | 5 | 3 | 3 | 3 | 3 | 3 | 3 | 3 | 10 |

| | | | | | | | | | | |
|---|---|---|---|---|---|---|---|---|---|---|
| 1263 | 1 | 1 | 3 | 3 | 3 | 1 | 3 | 3 | 3 | 9 |
| 1266 | 1 | 4 | 3 | 3 | 3 | 3 | 3 | 3 | 3 | 7 |
| 1275 | 1 | 3 | 3 | 3 | 3 | 3 | 3 | 3 | 3 | 6 |
| 1278 | 2 | 4 | 3 | 3 | 3 | 3 | 3 | 3 | 3 | 4 |
| 1280 | 1 | 0 | 3 | 3 | 3 | 3 | 3 | 3 | 3 | 7 |
| 1281 | 1 | 3 | 3 | 3 | 3 | 3 | 3 | 3 | 3 | 1 |
| 1283 | 1 | 2 | 1 | 0 | 0 | 1 | 2 | 1 | 1 | 4 |
| 1285 | 1 | 2 | 3 | 3 | 3 | 3 | 3 | 3 | 3 | 7 |
| 1286 | 1 | 5 | 3 | 2 | 2 | 3 | 3 | 3 | 3 | 6 |
| 1288 | 1 | 0 | 2 | 3 | 3 | 3 | 3 | 3 | 3 | 2 |
| 1293 | 1 | 3 | 2 | 0 | 2 | 3 | 2 | 2 | 2 | 5 |
| 1294 | 1 | 0 | 2 | 3 | 3 | 3 | 3 | 3 | 3 | 6 |
| 1295 | 1 | 2 | 3 | 3 | 3 | 3 | 3 | 3 | 3 | 5 |
| 1299 | 1 | 5 | 3 | 2 | 3 | 3 | 3 | 3 | 3 | 7 |
| 1301 | 1 | 2 | 3 | 3 | 3 | 3 | 3 | 3 | 3 | 10 |
| 1304 | 1 | 3 | 3 | 3 | 3 | 3 | 3 | 3 | 3 | 7 |
| 1306 | 1 | 1 | 3 | 3 | 3 | 3 | 3 | 3 | 3 | 8 |
| 1308 | 1 | 2 | 3 | 3 | 3 | 3 | 3 | 3 | 3 | 5 |
| 1309 | 3 | 2 | 3 | 3 | 3 | 3 | 3 | 3 | 3 | 10 |
| 1311 | 1 | 3 | 2 | 2 | 3 | 2 | 2 | 2 | 3 | 8 |
| 1317 | 1 | 5 | 3 | 3 | 3 | 3 | 3 | 3 | 3 | 5 |
| 1322 | 1 | 2 | 3 | 3 | 3 | 3 | 3 | 3 | 3 | 4 |
| 1324 | 1 | 3 | 3 | 3 | 3 | 3 | 3 | 3 | 3 | 3 |
| 1327 | 1 | 1 | 3 | 3 | 3 | 3 | 3 | 3 | 3 | 1 |
| 1330 | 1 | 2 | 2 | 1 | 0 | 0 | 2 | 0 | 1 | 4 |
| 1331 | 1 | 3 | 3 | 3 | 3 | 3 | 3 | 3 | 3 | 10 |
| 1334 | 2 | 8 | 3 | 3 | 3 | 3 | 3 | 3 | 3 | 10 |
| 1344 | 1 | 1 | 3 | 3 | 3 | 3 | 3 | 1 | 1 | 4 |
| 1345 | 1 | 3 | 3 | 3 | 3 | 3 | 3 | 3 | 3 | 6 |
| 1349 | 1 | 4 | 3 | 3 | 3 | 3 | 3 | 3 | 3 | 6 |
| 1355 | 1 | 2 | 3 | 3 | 3 | 3 | 3 | 3 | 3 | 8 |
| 1362 | 1 | 2 | 3 | 3 | 3 | 3 | 3 | 3 | 3 | 9 |
| 1365 | 2 | 4 | 3 | 3 | 3 | 3 | 3 | 3 | 3 | 7 |
| 1369 | 2 | 4 | 3 | 3 | 3 | 3 | 3 | 3 | 3 | 7 |
| 1370 | 1 | 1 | 3 | 3 | 3 | 3 | 3 | 3 | 3 | 4 |
| 1372 | 1 | 1 | 3 | 3 | 3 | 3 | 3 | 3 | 3 | 6 |
| 1373 | 1 | 6 | 3 | 3 | 3 | 3 | 3 | 3 | 3 | 1 |
| 1377 | 1 | 2 | 3 | 3 | 3 | 3 | 3 | 3 | 3 | 10 |
| 1378 | 2 | 3 | 3 | 3 | 3 | 3 | 3 | 3 | 3 | 10 |
| 1381 | 1 | 1 | 3 | 3 | 3 | 3 | 3 | 3 | 3 | 8 |
| 1384 | 1 | 3 | 2 | 3 | 3 | 3 | 3 | 3 | 3 | 6 |
| 1387 | 1 | 2 | 3 | 2 | 3 | 3 | 3 | 3 | 3 | 3 |
| 1388 | 2 | 1 | 3 | 1 | 3 | 2 | 1 | 2 | 3 | 9 |
| 1391 | 1 | 3 | 2 | 1 | 3 | 3 | 3 | 3 | 3 | 2 |
| 1392 | 3 | 4 | 3 | 3 | 3 | 3 | 3 | 3 | 3 | 9 |
| 1396 | 1 | 2 | 3 | 3 | 3 | 3 | 3 | 3 | 3 | 8 |
| 1399 | 1 | 4 | 3 | 3 | 3 | 3 | 3 | 3 | 3 | 8 |
| 1400 | 1 | 2 | 3 | 3 | 3 | 3 | 3 | 3 | 3 | 8 |
| 1401 | 1 | 2 | 3 | 3 | 3 | 3 | 3 | 3 | 3 | 5 |
| 1407 | 1 | 1 | 3 | 3 | 3 | 3 | 3 | 3 | 3 | 8 |

| | | | | | | | | | | |
|---|---|---|---|---|---|---|---|---|---|---|
| 1410 | 1 | 9 | 3 | 3 | 3 | 3 | 3 | 3 | 3 | 9 |
| 1412 | 1 | 7 | 3 | 3 | 3 | 3 | 3 | 3 | 3 | 7 |
| 1413 | 1 | 3 | 3 | 3 | 3 | 3 | 3 | 3 | 3 | 1 |
| 1416 | 1 | 5 | 3 | 3 | 3 | 3 | 3 | 3 | 3 | 2 |
| 1417 | 1 | 5 | 2 | 3 | 3 | 3 | 3 | 3 | 2 | 6 |
| 1419 | 1 | 3 | 3 | 3 | 3 | 3 | 3 | 3 | 3 | 5 |
| 1420 | 2 | 7 | 3 | 3 | 3 | 3 | 3 | 3 | 3 | 10 |
| 1421 | 2 | 3 | 3 | 3 | 3 | 3 | 3 | 3 | 3 | 7 |
| 1422 | 1 | 1 | 3 | 3 | 3 | 3 | 3 | 3 | 3 | 9 |
| 1424 | 1 | 1 | 3 | 3 | 3 | 3 | 3 | 3 | 3 | 4 |
| 1428 | 1 | 2 | 3 | 3 | 3 | 3 | 3 | 3 | 3 | 8 |
| 1431 | 2 | 5 | 3 | 3 | 3 | 3 | 3 | 3 | 3 | 8 |
| 1432 | 2 | 3 | 2 | 3 | 3 | 3 | 3 | 3 | 3 | 8 |
| 1435 | 2 | 3 | 3 | 3 | 2 | 2 | 3 | 3 | 2 | 6 |
| 1436 | 2 | 3 | 3 | 3 | 3 | 1 | 1 | 0 | 3 | 8 |
| 1437 | 2 | 3 | 3 | 3 | 3 | 3 | 2 | 3 | 3 | 8 |
| 1438 | 2 | 1 | 3 | 3 | 3 | 3 | 3 | 3 | 3 | 11 |
| 1439 | 2 | 2 | 3 | 3 | 3 | 3 | 3 | 3 | 3 | 10 |
| 1442 | 2 | 6 | 1 | 2 | 2 | 2 | 1 | 3 | 1 | 8 |
| 1443 | 1 | 1 | 3 | 1 | 2 | 1 | 3 | 0 | 2 | 8 |
| 1444 | 1 | 8 | 3 | 3 | 3 | 3 | 3 | 3 | 3 | 6 |
| 1445 | 1 | 2 | 3 | 3 | 3 | 3 | 3 | 3 | 3 | 2 |
| 1449 | 1 | 2 | 3 | 3 | 3 | 3 | 3 | 3 | 3 | 4 |
| 1450 | 2 | 2 | 3 | 3 | 3 | 3 | 3 | 3 | 3 | 7 |
| 1452 | 1 | 1 | 3 | 2 | 2 | 3 | 3 | 3 | 3 | 9 |
| 1456 | 1 | 3 | 3 | 1 | 3 | 3 | 3 | 3 | 3 | 4 |
| 1458 | 2 | 3 | 3 | 1 | 3 | 2 | 3 | 0 | 3 | 7 |
| 1460 | 1 | 1 | 3 | 3 | 3 | 3 | 3 | 3 | 3 | 5 |
| 1462 | 1 | 1 | 2 | 0 | 2 | 2 | 2 | 2 | 2 | 8 |
| 1463 | 2 | 1 | 2 | 2 | 2 | 2 | 2 | 2 | 2 | 3 |
| 1475 | 1 | 2 | 2 | 2 | 2 | 3 | 3 | 3 | 3 | 9 |
